# The Story of the Insane from Year to Year

**Published:** 1906-03-31

**Authors:** 


### THE STORY OF THE INSANE FROM YEAR TO YEAR.

Eighteenth Annual Series.

### THE DERBY COUNTY ASYLUM.

On January 1st this Asylum contained 766 patients, and on December 31st the number had fallen to 750. There were 183 first admissions; 26 readmissions; 74 discharged recovered; 16 discharged relieved; 30 discharged not improved; and 104 deaths. The average number resident during the year was 758, and the total number under treatment was 975. Calculated on the admissions, the recoveries stand at 37.5 per cent., and calculated on the average number resident, the deaths stand at 13.7. The first of these percentages is about the average in kindred institutions, and the latter is above the average. Of the year's deaths, 25 were caused by cerebral and spinal diseases; 36 by consumption; 11 by pneumonia; two by other lung diseases; and three by dysentery. The chief causes of the insanity in the year's admissions were : Moral causes, which collectively account for 43; intemperance in drink for 24; hereditary influence for 56; previous attacks for 31; and in 23 no cause was ascertained. Dr. Legge must have had a very anxious time during the year to which the report refers, for there were six rather serious, though non-fatal casualties, there were five inquests held by the Coroner, and "an exceptional number of suicidal and troublesome patients have been in residence." This is a peculiarity often found in asylums. One year will produce a crop of quite unpreventible incidents, to be followed, fortunately, by several years of comparative calm. To add to the trouble there were 31 cases of dysentery, 11 of influenza, and 16 of cellulitis among the patients; and 15 cases of influenza, one case of measles, one of scarlatina, one of erysipelas, and two of cellulitis among the staff. It is only someone familiar with the inner life of asylums who can realise what all this means to the medical staff. In this Asylum, also, the assistant medical officers train the nursing staff in their duties; and nine nurses and six attendants obtained the ordinary nursing certificates. We are not told whether there is a complete three years' course, or whether the Committee grant higher pay to those who hold the certificate. Three members of the staff received pensions during the year. One of these was Attendant Jonathan Smith, who had been attendant for 28 years. The weekly charge to the Union is 10s. 0¾d. "The Committee desire to place on record their high appreciation of the services of the Medical Superintendent and staff."

